# Institutional Needs

**Published:** 1914-03-28

**Authors:** 


### Institutional Needs.

### A NOVEL ADJUNCT TO THE POISON CUPBOARD.

While it is true to say that every institution will continue to require a cupboard, or repository, where poisons and dangerous drugs must be kept, the various shapes of bottles recommended so often for different substances of late years suggest that a poison cupboard in itself is not always sufficient. A specimen of a cork which locks up the bottle in which it is inserted has been sent to us from Mr. H. Yeild Collins, 4 Market Street, Stourbridge, and, though the inventor designed it for fastening up individual bottles of potable spirits on the well-known "Tantalus" principle, his suggestion that the cork might be used with advantage for poison bottles is quite practical. On the top of the cork a metal disc is placed in which is fitted a bolt joining another disc of the same shape. The other disc and the bolt attaching it through the head of the cork are on the railway buffer principle, but with the difference that when the "buffer" is compressed it is locked automatically, and thus is absolutely flush with the neck of the bottle into which the cork is inserted. To withdraw the buffer so that a purchase may be obtained for pulling out the cork a key, like a skeleton "Yale" in appearance, is inserted, and on pressing it home out springs the outer disc. Thus grasped the cork may be pulled out easily. This wonderfully ingenious contrivance, of which we believe the cost is slight, not only locks up every bottle so effectually that apparently nothing but a key can unlock it again, but also indicates, from this precaution, that the contents are not to be handled with impunity. The "Tantalus" Bottle Lock Cork has possibilities for hospitals and institutions well worth putting to the test. But is the key strong enough?

